# Eosinophilic cystitis refractory to steroids successfully treated with benralizumab: A case report

**DOI:** 10.3389/falgy.2022.1055129

**Published:** 2023-01-10

**Authors:** George N. Konstantinou, Vasiliki Voukelatou

**Affiliations:** Department of Allergy and Clinical Immunology, 424 General Military Training Hospital, Thessaloniki, Greece

**Keywords:** asymptomatic, biologics, eosinophilia, hematuria, dysuria, hypereosinophilic syndrome, urgency, pelvic pain

## Abstract

We report a case of a 66-year-old male diagnosed with refractory to oral corticosteroids eosinophilic cystitis (EoC). Hematuria was the first and only sign of the disease that was otherwise asymptomatic, and the only abnormal lab finding he had was peripheral eosinophilia (700 cells/*μ*l). Due to cardiovascular issues, an invasive surgical procedure was declined. As an alternative, benralizumab, an anti-IL-5R*α* monoclonal antibody with anti-eosinophilic properties, was administered. The patient responded rapidly with clinical and histological complete remission of the EoC four months after benralizumab started. He continued benralizumab 30 mg Q4-weeks for 12 months without experiencing any side effects. Six months after the last dose, he is completely healthy with no peripheral eosinophilia. EoC is a rare condition with no standardized treatment. Those with corticosteroid-refractory EoC are eligible for surgery. Benralizumab has an excellent safety profile; therefore, it should be considered before deciding on invasive surgical procedures in selected, refractory to non-specific treatment cases, especially with EoC of unclear etiology. It is unclear if benralizumab may immunomodulate the unknown underlying mechanisms of EoC, considering that EoC did not relapse after benralizumab was deemed eliminated. Further studies are needed to investigate this possibility.

## Introduction

Eosinophilic cystitis (EoC) is a rare inflammatory disorder of the bladder. Histologically it is characterized by eosinophilic infiltration of the bladder wall. Fibrosis and muscle necrosis could also be found in some cases. Allergens, medications (e.g., intravesical instillation and chemotherapeutic agents), parasitic infections, bladder carcinomas, bladder injury, and hypereosinophilic syndrome have been postulated as triggers or associated factors/conditions. However, the etiology of EoC remains unclear. The clinical presentation of the disease usually includes dysuria (any discomfort associated with urination), irritative voiding, urinary frequency, urgency (sudden, often unbearable need to urinate), hematuria, and suprapubic pain (1).

Most patients are treated with oral corticosteroids, but other non-specific medications such as antihistamines, nonsteroidal anti-inflammatory drugs, and leukotriene antagonists have also been used. In cases unresponsive to medication, invasive procedures (e.g., transurethral resection, partial or total cystectomy, and augmentation ileocystoplasty) are among the few alternative therapeutic options (2).

Monoclonal antibodies targeting IL-5 (mepolizumab, reslizumab) and the alpha subunit of the IL-5 receptor (IL-5R*α*), (benralizumab), have already been approved for the treatment of severe eosinophilic asthma and are promising agents for the treatment of other eosinophil-mediated disorders (3). In particular, benralizumab is a humanised afucosylated, monoclonal antibody that selectively binds to the IL-5R*α* expressed on the surface of eosinophils and basophils. The removal of fucose in the Fc domain of this IgG1 anti-IL-5R*α* antibody increases its affinity for the FcɣRIII receptors on immune effector cells like natural killer cells. This results in eosinophils apoptosis through enhanced antibody-dependent cell-mediated cytotoxicity (ADCC), leading to eosinophil-depleting activity (benralizumab Summary of Product Characteristic) (4).

## Case report

We present a case of a 66-year-old man referred to our Allergy Department for peripheral eosinophilia and a diagnosis of EoC. Informed consent was obtained from the patient to publish his history details and relevant figures.

The presented history started in December 2017. At this time, the patient reported asymptomatic, macroscopic hematuria for the first time in his life. A cystoscopy at that time revealed a small polyp on the posterior bladder wall. A trans-urethral cauterization was performed. Macroscopically no other obvious pathology was found apart from benign, mild prostatic hypertrophy.

On February 2020, the patient had a bovine aortic valve replacement and began clopidogrel bisulfate 75 mg qd, bisoprolol hemifumarate 2.5 mg qd, and rosuvastatin calcium 10 mg qd.

The patient remained free of macro- or microscopic hematuria or any symptoms until July 2020, when there was a relapse with massive hematuria. Cystoscopy showed a large bladder mass, which appeared as a solitary tumor-like lesion originating from the urothelium with a wide base and surrounding inflammation at the posterior bladder wall. Multiple biopsies were performed followed by cauterization. Histologically, there was edema, epithelial cell apoptosis, squamous metaplasia, and prominent, dense inflammatory infiltration of eosinophils under the surface of the urothelium (>200 eosinophils/HPF). There was no histopathological evidence of neoplasia. Biopsies were followed by a whole-body CT scan that demonstrated bladder wall thickening, a large calcified echinococcus cyst in the spleen, known since he was ten years old, and osteoarthritis of both knees and the first metatarsophalangeal joints. All his urine cultures were negative.

Three faecal samples, collected over 14 days, and serology tests for parasites, including *Echinococcus granulosus*, *Toxocara canis*, and *Trichinella spiralis*, were negative. Examination for autoimmune disorders was negative as well. Esophagoscopy, gastroscopy, duodenoscopy, and colonoscopy were additionally performed and were interpreted as negative for any obvious process, along with relevant random biopsies excluding eosinophilic gastrointestinal disorders.

**Figure 1:**
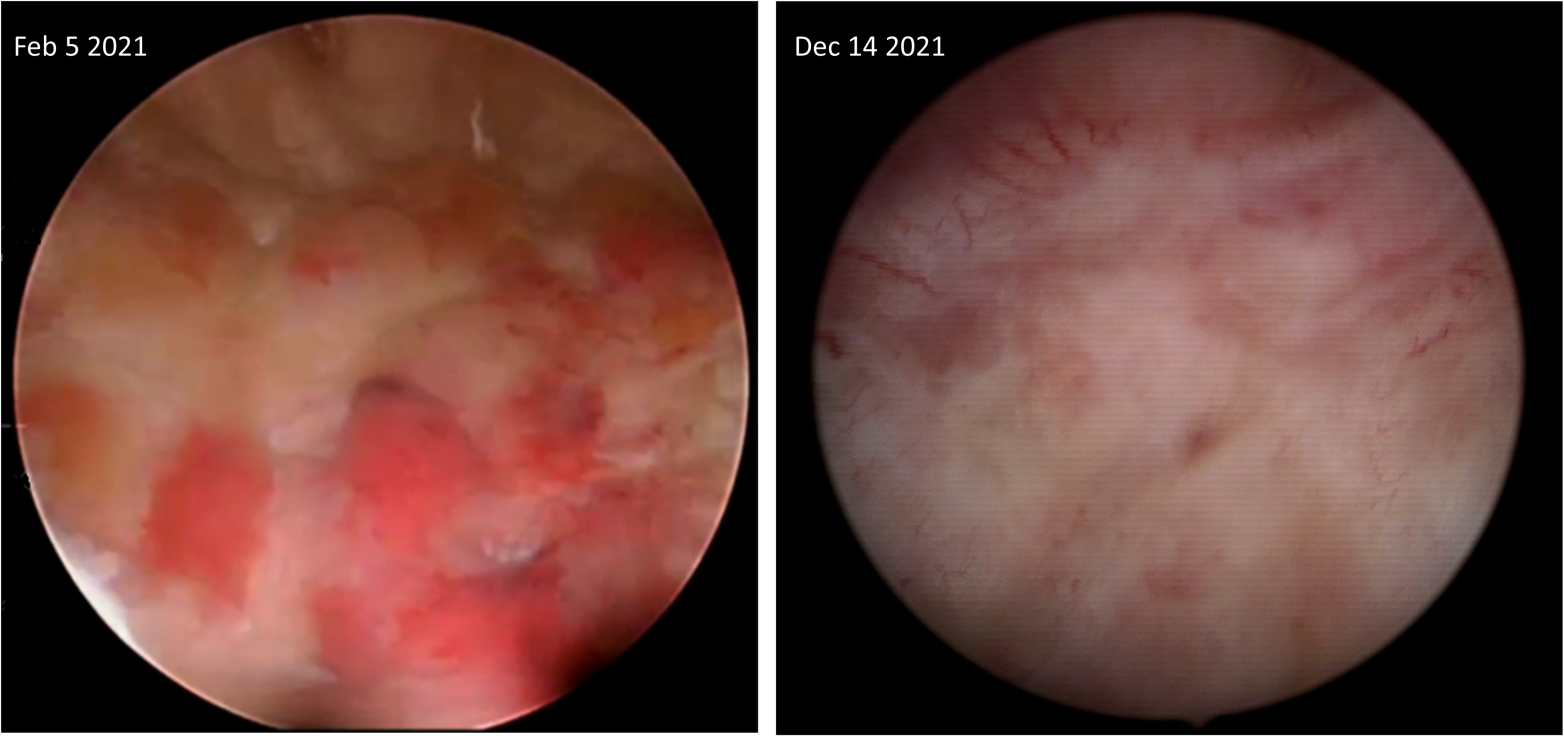
Cystoscopic images depicting part of the affected posterior bladder wall before starting and four months after being on benralizumab.

The patient started an empirical treatment with levocetirizine 5 mg qd. Until September 2020, he relapsed twice with hematuria that lasted less than 24 h. On February 5th, 2021, he had another massive hematuria causing a hematocrit drop from 38% to 29%. The urologists performed a transurethral resection of the lesion (Figure 1), confirming the diagnosis of EoC. At that point, antihistamines stopped, and methylprednisolone 8 mg qd was started.

Unfortunately, the patient had severe hematuria a few months later, on May 31st, 2021, with endoscopic evidence of relapse of the tumor-like lesion, after which he was referred to us.

He did not report any personal or family history of allergies. The patient had peripheral eosinophilia (700 cells/μl), normal total IgE (14.3 IU/ml), and serum tryptase levels (5.2 μg/L). The specific IgE levels and the skin prick tests for common environmental and major food allergens were also negative. Fluorescent *in situ* Hybridization (F.I.S.H) was performed, and deletion or translocation of genes involved in hypereosinophilic syndrome [FIP1L1-CHIC2-PDGFRA (4q12), PDGFRB (5q32-q33.1)] were not detected.

The patient was an ex-smoker who overconsumed green tea for the last 30 years when he started tea trading. He reported no exposure to any chemical substance and was not taking any vitamin supplements regularly.

Because of the need to be on anticoagulant medication, surgical intervention was not considered the best alternative. Considering benralizumab as a specific anti-eosinophilic medication, we decided on the off-label use of benralizumab after getting informed consent from the patient and approval from the Greek National Organization for Medicines Committee (approval numbers: *ΔΒ*4*Α*/*Γ*31/293, *ΔΒ*4*Α*/*Γ*31/273). Methylprednisolone was stopped because his EoC was refractory to steroids. The patient was given benralizumab 30 mg subcutaneously every 4 weeks starting from July 2021 and followed up with a urinalysis once a month.

Microscopic hematuria disappeared after the second dose. After five doses, a cystoscopy with an additional biopsy were performed, demonstrating complete endoscopic (Figure 1) and microscopic remission. Moreover, the peripheral eosinophil level decreased to 0 cells/μl. The patient continued benralizumab for 12 months. He received the last dose on June 2022. Until now (updated on December 4th, 2022), the patient is symptom-free, with normal peripheral eosinophils (72 cells/μl) without macro- or microscopic hematuria. No adverse effects related to benralizumab were reported.

## Discussion

This is one of the few cases with EoC with the additional extremely rare feature among them of being asymptomatic. Hematuria was the first and only sign of the disease. The disease was refractory to steroids but was treated successfully with benralizumab. There is neither a standard dose scheme nor a specific treatment duration for EoC with benralizumab, as there is no relevant experience. The Greek National Organization for Medicines Committee decided the duration of 12 months based on the rapid response with clinical and histological remission within four months. The recommendation was to closely and regularly follow up the patient for at least two years. In the case of EoC relapse, the re-administration of benralizumab will be re-examined.

There are two cases successfully treated with benralizumab in the literature, but both had symptomatic EoC. The first such case was a 78-year-old woman experiencing extreme dysuria, urgency, frequency, incontinence, pelvic pain, and hematuria, which were unresponsive to various pharmacologic treatments. The patient had a significant improvement in her symptoms and quality of life after initiating benralizumab, but not complete remission of EoC. After nine months of therapy, a biopsy showed continued cystitis but rare eosinophils (5).

In another case report, a 49-year-old man with symptomatic EoC was also offered benralizumab. He had a severe bladder wall thickening and, therefore, an inability to retain a sufficient volume of urine, leading to continuous leakage, dysuria, and pelvic pain. Treatment with benralizumab led to clinical and histological remission with no need for any surgical intervention, but the time needed was not reported (6).

EoC is a rare condition with no standardized treatment. Most patients respond to corticosteroids, but long-term use is linked with multiple adverse effects. Those with corticosteroid-refractory EoC are eligible for surgery. The advent of anti-eosinophil monoclonal antibodies like benralizumab gave new treatment options for patients with eosinophilic disorders. Our patient rapidly responded with clinical and histological improvement achieving complete remission of his EoC four months after benralizumab initiation. This effect, including the normalized peripheral eosinophil number, sustains after seven months without any medication. A tissue-related pharmacokinetic irregularity, including impaired steroid absorption, distribution, and metabolism in the area of the bladder wall where the eosinophil accumulation occurred may explain why he did not respond to steroids, but this has to be further investigated among steroid-refractory EoC cases.

Regarding side effects, we cannot be sure if we can extrapolate the eosinophil asthma experience to other eosinophilic disorders, including EoC (7). Our case did not experience any side effects that could be attributed to benralizumab. Based on the three relevant cases that have been published, benralizumab seems to have an excellent safety profile in EoC. Further studies with a sufficient number of patients are needed to prove the benralizumab excellent safety profile that our case showed. Until then, any effort to treat similar cases with benralizumab needs close and continued follow-up.

Therefore, benralizumab can be considered before deciding on invasive surgical procedures in selected refractory to non-specific treatment cases, especially with EoC of unclear etiology. It is unclear if benralizumab may immunomodulate the unknown underlying mechanisms of eosinophilic cystitis, considering that it eradicated macroscopically and microscopically the EoC pathology, an effect that sustained after benralizumab is deemed eliminated. Further studies are needed to investigate this possibility.

## Data Availability

The raw data supporting the conclusions of this article will be made available by the authors, without undue reservation.
